# Public Perceptions and Understanding of Risks Associated with Do-It-Yourself Orthodontics: A Survey Study

**DOI:** 10.4317/jced.59911

**Published:** 2022-09-01

**Authors:** Claudia Acosta-Lenis, Prashanti Bollu, Kishore Chaudhry, Richard Stevens, Karthikeyan Subramani

**Affiliations:** 1 Roseman University of Health Sciences, College of Dental Medicine, Henderson, NV, USA

## Abstract

**Background:**

The purpose of this study was to evaluate general public’s perception, knowledge and preferences on orthodontic treatment rendered by an orthodontist versus the “Do-It-Yourself” orthodontics (DIYO) concept without professional supervision. The secondary objective was to assess laypeople’s awareness on the risks and limitations of DIYO.

**Material and Methods:**

A 24-question online survey questionnaire was administered to 526 laypeople who had no professional experience or background in dentistry and orthodontics. All data was collected over 3 days (July 20-23, 2020) by Qualtrics server and forwarded to the principal investigator. Statistical analysis was done with statistical software SPSS® version 26.

**Results:**

The most important reason for laypeople to opt for DIYO is financial reason. People who have undergone orthodontic treatment know the difference between a general dentist and an orthodontist, whereas people who have not had orthodontic treatment are less likely to know the difference. Of the 285 people who did not receive orthodontic treatment before by a dental professional, 43 have considered DIYO. 122 of the 526 people considered DIYO, and 79 of the 122 had orthodontic treatment before. 26 of the 122 did not consider the clinical exam and diagnostic records important and would be comfortable without in-person supervision. 83 of the 122 would be comfortable not having in-person supervision, and still considered this treatment modality “Doctor-Directed”.

**Conclusions:**

The main reason laypeople utilize DIYO is the low cost. Some DIYO users do not consider risks involved and a small percentage consider their own dentist to be responsible if any issues arise with DIYO. One third of survey respondents will consider DIYO in the future.

** Key words:**Do-It-Yourself (DIY), Direct-To-Consumer (DTC), Adult, Orthodontics, Dentistry, Surveys and Questionnaires.

## Introduction

Orthodontic treatment requires comprehensive, patient-oriented clinical evaluation performed by a trained professional (Orthodontist). Different treatment modalities are often required to correct the underlying malocclusion. Continuous evaluation and supervision of treatment by the orthodontist is mandatory for an ideal treatment outcome. The Do-It-Yourself (DIY) concept has a long history in the USA, especially in the domain of home renovation, but in general it is applied to any method involving construction, reconstruction, or repairing things without the direct assistance of a professional in a given field. Regarding the dental profession, it is important to note that DIY products and services are not provided by licensed dentists or specialists and are not consistent with the Dental Practice Act of all 50 states in the USA. Taking high-quality impressions, for example, requires time and training, and a failure in this step can lead to errors in the proper diagnosis and treatment (1). Moreover, studies have demonstrated that orthodontists spend more time on treatment planning and achieving better quality treatment outcomes than general practitioners who have not undertaken an extensive training of specialization in orthodontics (2).

Recently, the Do-It-Yourself orthodontics (DIYO) concept has emerged as an alternative for general public (laypeople) to treat malocclusions by themselves, with an online alliance. DIYO refers to a patient’s self-directed efforts to move teeth without the orthodontist’s supervision, and bypassing important diagnostic means (3). This philosophy is also known as “Direct-to-Customer” (DTC), “Doctor- Directed,” and “At Home Clear Aligner Therapy”. The label “doctor-directed” is misleading and the difference between DIYO and DTC is minimal. The first report of DIYO was published in 2016 and it reported the case of a 23-year-old student at New Jersey Institute of Technology who, after researching the literature on aligners, took his own alginate impressions, poured them up with PermaStone® (Galeton, PA), scanned the casts, and then employed a software to digitally model tooth alignment towards what he determined was the proper position (4,5). After buying specific plastic material on the internet, he fabricated 12 aligners over the same number of models he created using a 3D printer. The reported total cost of treatment was $60 (4,5).

After analyzing the economic implications of the first DIYO report, entrepreneurs rapidly capitalized on the idea. DIYO companies drafted a model in which self-taken photos and self-taken impressions using mold kits (or an intraoral scan taken in one of their DIYO shops) are the only requirements. Medical/dental history, physical examination, and diagnostic records are not part of the equation. DIYO companies hold the client responsible for seeking dental care before and after treatment. The client completes a questionnaire and signs the informed consent and arbitration agreements. When a layperson buys DIYO, a dentist or an orthodontist is notified by email that the customer’s treatment plan is ready for review. Treatment is only targeted at aligning the anterior teeth over the course of a few months, with the user receiving new sets of aligners by mail. The customer self-evaluates the results, which means that treatment is not “doctor-directed.” Furthermore, comprehensive records, treatment objectives, treatment plan/alternatives, normal and abnormal clinical findings, description of the treatment rendered, proper informed consent, referrals, and other important considerations are not part of DIYO. From a legal standpoint, these missing documents are the only way an orthodontist can support decisions and interactions during the treatment (6). As of October 2021, there were at least five companies offering at-home aligners in the USA (7) and seven companies in the UK (8). By eliminating in-person professional supervision and monitoring, companies are able to offer treatment for thousands of dollars less (7). A recent study which evaluated the quality of information contained within the websites of DTC orthodontic aligner providers concluded that quality of information contained within the websites is poor (9). The reason why laypeople choose DIYO is not reported in the literature. No study has been conducted on assessing on the knowledge of laypeople on DIYO. The primary purpose of this study was to evaluate laypeople preferences and knowledge of orthodontic treatment done by an orthodontist versus DIYO. The secondary objectives were to examine the reasons laypeople utilize DIYO and to establish if they were aware of the risks and limitations involved.

## Material and Methods

To fulfill the aims of the present investigation, an observational survey study was designed and implemented. This study was approved by the Roseman University of Health Sciences Institutional Review Board: [1538015-4]. A survey questionnaire with 24 questions was developed by the authors of this study. A pilot survey was done and a cover letter discussing the aims of the study were distributed by an online survey platform (www.qualtrics.com). Assuming a prevalence rate of 25% about knowledge of existence of DIY orthodontics, with a margin of error of 5 percentage points around the assumed prevalence, and 99% confidence level, the calculated sample size was 498. Inclusion criteria consisted of: Respondents 18 years to 65 years of age, persons without professional or specialized knowledge in the dental field and no dental education, training, or work experience. Dental students, dentists, hygienists, assistants, dental personnel were specifically excluded. Similarly, anyone with previous formal dental training were also excluded from the study. The survey was designed in a manner that, if the respondents who positively answered the question: “Have you ever considered doing Orthodontic treatment by yourself?” would continue the survey till the end. The survey questions were designed to: 1) Establish demographics, 2) Assess respondent’s familiarity with orthodontics, and 3) Assess respondent’s perception and understanding of risks of DIYO. Qualtrics informed us that they had a huge database of emails of persons over 18 years of age in United States, and the database is continuously growing. Qualtrics was asked to stop the survey once the desired sample size was achieved. Data was collected over three days and responses from 526 persons were received. The collected data was analyzed with IBM® SPSS® version 26. The major dependent variable was knowledge about existence of DIY orthodontics. Information on intentions and actual use of DIY orthodontics was also collected and analyzed. Association between descriptive variables was assessed by using Chi square test.

## Results

The survey received 526 completed responses. Gender-wise it was 50% females and 49% males. Millennials were 57% of the total sample. When analyzing annual household income, half of the sample earned less than $50,000, about 27% of the sample earned between $50,000 and $100,000. 76% of the sample earned less than $100,000. Although the education response was individual and the income in this survey was household based, findings were statistically significant (*p* < .001). Income, therefore, correlates highly with level of education.

-Laypeople’s familiarity with dentistry

**Figure 1:**
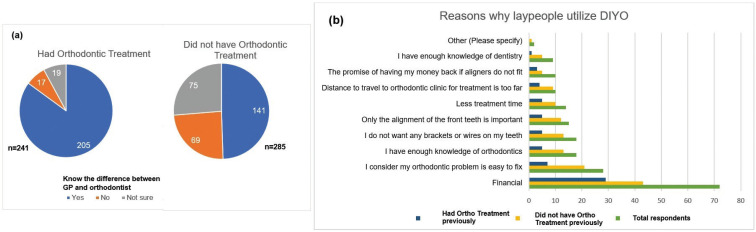
a. Awareness of the difference between GP and an orthodontist correlates with having prior treatment experience and who provided the treatment (n=526 respondents). (*p* < .001). b. Reasons why laypeople utilize DIYO. (Multiple responses were allowed).

In the second part of the survey, it was assessed if laypeople knew the difference between a general dentist and an orthodontic specialist. As far as previous history of orthodontic treatment, 241 (45.8%) out of 526 respondents had received orthodontic treatment (brackets, clear trays/aligners), while 285 (54.2%) had not received orthodontic treatment (Fig. 1a). Of the 241 people who answered yes to having received orthodontic treatment, 205 of them responded knowing the difference between a general practitioner (GP) and an orthodontist. Knowledge on difference between a GP and an orthodontist was higher among persons who had received orthodontic treatment (Fig. 1a). Among those who had orthodontic treatment and knew the difference between GP and an orthodontist, 93% of them were able to recognize the provider. 68% said the treatment was done by an orthodontist and 25% by a GP. These findings were statistically significant (*p* < 0.001).

-DIY and laypeople’s awareness

The last and most important part of this survey was to gather information about laypeople’s desire to utilize DIYO. 404 (76.8%) out of the 526 respondents have never considered DIYO, while 122 (23.2%) considered using it. Among the 122 respondents, 63 (51.6%) of them have used clear aligners, 51 (41.7%) of them have used rubber bands, and 8 (6.6%) of them used other devices. Among the 241 people who received orthodontic treatment (brackets, clear trays/aligners) by a dental professional, 79 (32.8%) of them have considered DIYO as well; these findings were statistically significant (*p* < .001). Of the 285 people who have not received orthodontic treatment, 43 (15.1%) have considered DIYO and 242 (84.9%) of them have not considered DIYO.

We asked laypeople the reasons why they would consider utilizing DIYO. We chose the following three groups: the whole population (526 respondents), those who previously had orthodontic treatment done by a professional (241), and those who never had orthodontic treatment done by a professional (285). All three groups had 3 main reasons in common for considering DIYO, financial being the primary reason (Fig. 1b). The second most common reason was that they considered their malocclusion easy to fix. The third most common reason was divided between a desire to avoid having braces or wires on their teeth, and people thinking that they had sufficient knowledge of orthodontics to manage their own treatment without professional supervision. Reduced treatment time and the promise of having their money refunded were two additional reasons given by the 285 who never had orthodontic treatment before. For two of these groups, the total population, and those who had received treatment before, the fourth most common reason was that they only considered alignment of the front teeth to be important. For the total population, other common reasons were divided among reduced treatment time, elimination of travel, the promise of getting their money back if the aligners did not fit and believing that they had enough knowledge in orthodontics. For the 241 people who received orthodontic treatment previously, the other common reasons were eliminating the need to travel to the dental office, less treatment time, having enough knowledge of dentistry, and the promise of having the money back if the aligners did not fit. Among the 285 people who had not received orthodontic treatment, the fourth most common reason was having enough knowledge in dentistry. The fifth most common reason was believing that only the alignment of the front teeth was important. The distance to travel to the dental office was not considered a reason for utilizing DIYO. The main reason to opt for DIYO was financial. Surprisingly enough, the reasons people who had considered DIYO were basically in the same order for people who had received orthodontic treatment before and those who had not. Of the total population, almost a half of them had an annual household income of less than $100,000.

When laypeople were asked if they thought there were some risks involved in orthodontic treatment, 244 answers were received. Among the 122 respondents who would consider DIYO, the answers were as follows: the first most common risk was tooth mobility/loose teeth, the second was the loss of tooth support/bone loss. Third was that it will not straighten the teeth, the fourth was receding gums/gum disease, the fifth was making overbite worse, and the least most common risk perceived was that orthodontic treatment would make the front teeth stick out. 18 people did not consider any of these as potential risks involved in orthodontic treatment.

**Figure 2:**
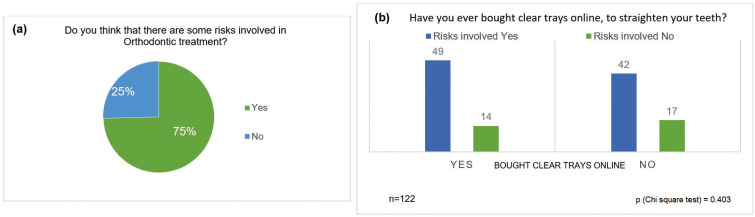
a. Awareness of risks involved in Orthodontic Treatment. b. Assessment of risks involved for those who bought clear trays online.

From this point, the survey focused on the group of 122 respondents who answered positively to considering DIYO. Of those 122 people, 91 (75%) thought there were some risks involved in orthodontic treatment, in contrast to 31 (25%) who did not think there were risks involved (Fig. 2a). 63 (51.6%) out of the 122 people who had considered DIYO had bought clear trays online to straighten their teeth, 49 (77.8%) of the 63 thought there were some risks involved, while 14 (22.2%) of the 63 did not think there were risks involved. However, this was not statistically significant (*p*=0.403), as shown in Fig. 2b.

**Figure 3:**
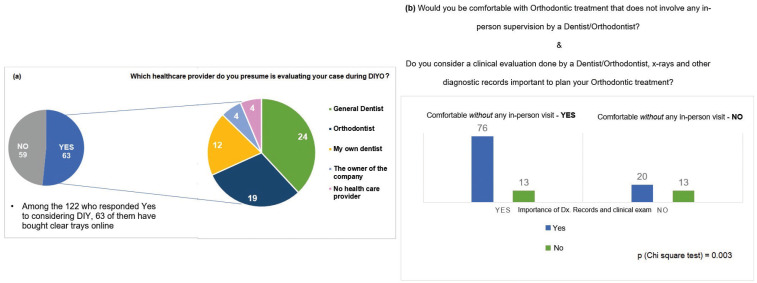
a. Awareness of what healthcare provider lay people presume is evaluating their case while DIYO. b. Importance of diagnostic records and comfort without any in-person supervision by dental professional.

Among the 63 people who bought clear aligners online, 24 of them assumed a GP was evaluating their case, 19 of them assumed an orthodontist was evaluating, 12 of them assumed their own dentist was evaluating, 4 of them assumed the owner of the company was evaluating, and finally 4 more assumed no health care provider was evaluating the case (Fig. 3a). When the 122 people who considered DIYO were asked if they considered a clinical evaluation done by a Dentist/Orthodontist, x-rays, and other diagnostic records important to plan their orthodontic treatment, 96 (78.7%) people responded positively. 26 (21.3%) out of the 122 did not consider the clinical evaluation, x-rays, and other diagnostic records important to plan the orthodontic treatment. 54 of the 63 considered clinical evaluation important. 21 of the 54 presumed that a GP evaluated their case, 17 by an orthodontist, 11 by their own dentist, 3 by the owner of the company and 2 by a non-health care provider. Nine of the 63 respondents did not consider clinical evaluation to be important. 3 of them presumed that their case was evaluated by a GP, 2 by an orthodontist, 2 by non-health care provider, 1 by their own dentist and 1 by the owner of the company. However, we found this information to be not statistically significant (*p* < 0.27).

Eighty-nine out of the 122 people who responded yes to considering DIYO would be comforTable with orthodontic treatment that did not involve any in-person supervision by a dentist/orthodontist. In contrast, 33 of them would not be comforTable with no dentist/orthodontist supervision. 76 out of the 122 considered a clinical evaluation done by a dentist/orthodontist and diagnostic records to be important and would be comforTable with orthodontic treatment that does not involve any in-person supervision by a Dentist/Orthodontist. 13 of the 122 people did not consider a clinical evaluation done by a GP/orthodontist, x-rays and other diagnostic means important and would be comforTable with orthodontic treatment that did not involve any in-person supervision by a dentist/orthodontist. This was statistically significant (*p*=0.003), as shown in Fig. 3b.

**Figure 4:**
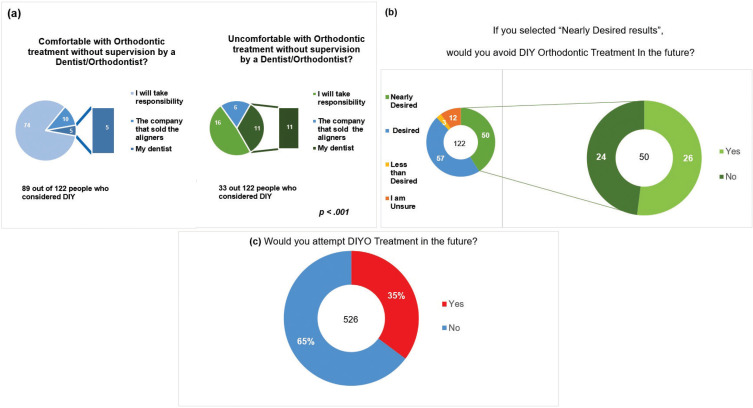
a. Laypeople assessment of responsibility and level of comfort without any in-person supervision by dental professional. b. Response of opting for or avoiding DIYO in respondents who expected nearly desired treatment results. c. 35% of the total respondents responded that they would attempt DIYO in the future.

In regard to who would be responsible for detecting issues or problems that may occur during DIYO, 90 (73.8%) out of the 122 respondents said they would take the responsibility, 16 (13.1%) would hold the company who sold the aligners responsible, and another 16 people (13.1%) responded that their dentist would be responsible. 122 people considered doing orthodontic treatment by themselves. 89 of them would be comforTable with orthodontic treatment without supervision by a dentist/orthodontist. 74 of the 89 (83.1%) will take responsibility for the issues or problems that may occur. 10 of the 89 (11.2%) would hold the aligner company responsible, and 5 of the 89 (5.6%) believed their own dentist to be responsible. Thirty-three people out of the 122 will not be comforTable with orthodontic treatment without supervision by a Dentist/Orthodontist. 16 of the 33 will be responsible for issues or any problems that may occur. 11 of the 33 would hold their own dentist responsible, and 6 of the 33 will hold the aligner company responsible. This was statistically significant (*p*< 0.001) (Fig. 4a). Nearly 10% of the people who expressed the desire to utilize DIYO are unsure of what results to expect, and 24 out of 50 who anticipated nearly desired results still considered utilizing DIYO (Fig. 4b). 35% of the 526 respondents will consider DIYO in the future (Fig. 4c).

## Discussion

Although patients have many motivations to seek orthodontic treatment, esthetics is by far the main reason to get orthodontic treatment done. In 2009, a study reported that parents (91.4%) and their children (93.4%) both graded esthetics as the primary reason to seek orthodontic treatment (10). More recently, Lin *et al*., demonstrated that the psychosocial impact of dental esthetics played an important role in the decision-making process of adults seeking orthodontic treatment (11). Since the esthetic region of the dentition is mainly the anterior zone, it would seem natural that someone would seek a relatively simpler mean to improve the esthetic zone. This tendency provides a convenient explanation for the appeal of DIYO, which focuses entirely on the anterior dentition. DIY/DTC companies advertise their products to be less expensive when compared to getting treated by an orthodontist. After analyzing the data gathered in our study, it was found that financial considerations were the first and most important reason for laypeople to opt for DIYO. Laypeople who have had orthodontic treatment actually know the difference between a GP and an orthodontist, whereas people who have not had orthodontic treatment are less likely to know the difference. Regarding the reasons why people are increasingly using DIYO, recent studies indicate that reduced cost is the main reason (12), which is in agreement with our results. This finding seems to be in harmony with laypeople feeling confident about performing DIYO, especially when pushed to do so by social media (13). For laypeople, who believe that they are fixing a problem, this actually means that the investment is minimal and that they believe they are savvy enough to pursue this course of action. They are not considering the inherent risks of their actions. In some cases, the consequences of DIYO can be devastating, as discussed by Froum *et al*. (14).

The clinical presentation of a given orthodontic problem or problems can be deceiving, which can lead to failures if incorrect diagnosis (or no diagnosis at all) and erratic treatment plans are designed as a consequence of poor medical/dental history taking. Orthodontic residents and orthodontists have been shown to have a more accurate assessment than other dental peers (15), which does not support laypeople’s self-perception on adequate or appropriate medical/dental/orthodontic knowledge. As for who provides orthodontic treatment and the reasons why laypeople opt to go to an orthodontist or utilize DIYO, a recent publication found that people with the highest level of interest in getting orthodontic treatment will look for an orthodontist, while those with the least interest prefer DIYO aligners (16). According to this study, the reason why people go to an orthodontist is quality of treatment. Our findings are in agreement with this study and laypeople opt for DIYO primarily due to costs and convenience, not quality of care. In agreement with Melsen (17), orthodontics is a patient-oriented profession that must distinguish between patients who need goal-oriented treatment with individually produced appliances. As the orthodontic profession has been witnessing the last few years with DIYO, due to the efforts and pressure of the market, patients are increasingly drawn into treatment by non-specialists with ‘‘Fast Food Orthodontics,” as DIYO might well be considered. Orthodontists must familiarize themselves with the products offered to the public by non-specialists, so patients can be effectively educated about new treatment modalities, their strengths, and their weaknesses, thus helping the patient make an informed decision.

As to who would be responsible if there was a problem during or after DIYO, 33.3% of respondents who were not comforTable with orthodontic treatment that did not involve any in-person supervision by a dentist/orthodontist would hold their own dentist responsible for any issues with DIYO. By signing the informed consent and the agreement to arbitrate, the client indicates that their dentist has performed a comprehensive dental exam and has determined that the patient is healthy from both a restorative and periodontal perspective (18). The question is: How are dentists responding to this? 

We as trained specialists should be proactive in informing the public about DIY health care activities that are potentially harmful (19). It may be appropriate to spend time educating the public and patients, and to clarify some misconceptions (18). One such misconception is risk. Interestingly, 22.2% of the people who bought clear trays online do not think that there are any risks involved. This study has explored the reasons and motives of laypeople opting for DIYO. The long-term effects of DIYO in terms of achieving esthetic and functionally stable occlusion, however, remain to be investigated.

## Conclusions

Within the limitations of this study, the following conclusions can be drawn:

• The main reason why laypeople utilize DIYO is low cost.

• Common characteristics among people who have considered DIYO include lower level of education, low income, lack of knowledge regarding the differences between a GP and an orthodontist. Women are more likely to utilize DIYO than men.

• 25% of potential DIYO users and buyers do not consider risks involved and a noticeable portion of them considered their own dentist responsible for detecting issues or problems during treatment.

• Some DIYO users believe there are no risks involved. Moreover, one third of respondents will consider DIYO in the future.
